# Gut microbiota regulates neuropathic pain: potential mechanisms and therapeutic strategy

**DOI:** 10.1186/s10194-020-01170-x

**Published:** 2020-08-17

**Authors:** Binbin Lin, Yuting Wang, Piao Zhang, Yanyan Yuan, Ying Zhang, Gang Chen

**Affiliations:** Department of Anesthesiology, Sir Run Run Shaw Hospital, School of Medicine, Zhejiang University, 3 Qingchun East Road, Zhejiang, 310016 Hangzhou China

**Keywords:** Gut microbiota, Neuropathic pain, Therapeutic strategy

## Abstract

Neuropathic pain (NP) is a sustained and nonreversible condition characterized by long-term devastating physical and psychological damage. Therefore, it is urgent to identify an effective treatment for NP. Unfortunately, the precise pathogenesis of NP has not been elucidated. Currently, the microbiota-gut-brain axis has drawn increasing attention, and the emerging role of gut microbiota is investigated in numerous diseases including NP. Gut microbiota is considered as a pivotal regulator in immune, neural, endocrine, and metabolic signaling pathways, which participates in forming a complex network to affect the development of NP directly or indirectly. In this review, we conclude the current understanding of preclinical and clinical findings regarding the role of gut microbiota in NP and provide a novel therapeutic method for pain relief by medication and dietary interventions.

## Introduction

Neuropathic pain is a sustained and nonreversible condition presenting pain as a direct aftereffect of a lesion or disease of the somatosensory system involving peripheral and central levels. It is typically chronic and frequently manifests as persistent or recurrent pain. Hence, the unpleasant feeling induces sleep, fatigue, and emotional disorders, and thereby results in an imbalance of work, leisure life, and family relationships. Epidemiological surveys showed that the prevalence of chronic pain with neuropathic characteristics is approximately 7–10% [1]. Additionally, 40% of patients went through some features of NP, referring to a survey of more than 12, 000 patients with both nociceptive and NP in Germany [2]. To date, accumulating evidence revealed that the occurrence and development of NP are implicated with peripheral and central sensitization, aberrant ectopic activity, pathological activation of microglia, and impaired inhibitory modulation [3]. Whereas, the underlying mechanism concerning NP are not fully understood, which causes the absence of effective treatments to alleviate pain substantially.

According to current estimates, approximately 10^14^ microbes are residing in the human body and the number of microbial cells is outnumbering the human cells [4]. In humans, the gastrointestinal tract is a huge, populous, and intricate microbial ecological community that mainly contains bacteria, archaea, fungi, protozoa, and viruses. Alteration of gut microbiota or unexpected exposure to specific bacteria in the intestine can regulate the peripheral and central nervous systems (CNS), leading to the change of brain function and illustrating the existence of the microbiota-gut-brain axis. It is now commonly believed that interaction in the microbiota-gut-brain axis is bidirectional. Excitingly, the interactive signal transmission has been proved to be involved in different kinds of diseases. Abundant work indicated that gut microbiota indeed plays a predominant role in the appearance of visceral pain and provides an infusive research interest in pathological pain linked to gut dysbiosis. The emerging role of gut microbiota in neurological diseases, including chronic pain, has attracted ever more traction recently.

Currently, the relationship between gut microbiota and pain modulation has attracted more and more clinicians’ attention along with the advancement of medical science. A growing body of research showed that bacteria could activate nociceptors directly via their products and constitutive elements [5]. During infection, bacterial formyl peptides induce calcium flux and action potentials in nociceptor neurons and thereby result in mechanical pain sensitivity in mice [6, 7]. Moreover, α-hemolysin, one of the pore-forming toxins secreted by *Staphylococcus aureus*, could induce neuronal firing and spontaneous pain [7, 8]. Interestingly, previous studies indicated that nociceptor neurons could especially recognize bacterial constitutive/secreted molecules, which are partly involved in the pain signaling [5, 9]. Besides, viral and fungal pathogens are identified to elicit alteration of pain sensitivity via inducing immune activation [10]. Additionally, there is no denying that microbes may serve as a critical and irreplaceable modulator in the progression of pain transduction according to previous researches.

Emerging evidence strongly demonstrated that gut microbiota plays a crucial role in abdominal pain, opioid tolerance, headache, inflammatory pain, and NP [11]. Among them, the connection establishment between gut microbiota and NP provides significant potential for researchers to overcome this type of refractory pain. Shen et al. investigated the role of gut microbiota in chemotherapy-induced peripheral neuropathy (CIPN) and confirmed that oxaliplatin-induced mechanical hyperalgesia was decreased in both germ-free (GF) mice and mice pretreated with antibiotics [12]. Accordingly, the protection would be abrogated after colonizing the microbiota in GF mice. Lately, a study established a rat model of spared nerve injury (SNI) and demonstrated that the anhedonia susceptible rats prefer to show gut microbiota dysbiosis when compared to sham-operated and resilient rats. Meanwhile, the transplantation of fecal microbiota from SNI rats to the pseudo-GF mice can also alter the severity of NP and the phenotypes of depression-like and anhedonia-like [13]. Although NP is rather difficult to treat and its mechanism remains unclear to date, increasing studies suggest that gut microbiota may be a promising target for improving NP management.

We comprehensively retrieved the PubMed database from 2000 to August 2020 and the retrieved keywords mainly consist of ‘neuropathic pain’ AND ‘gut microbiota’, ‘neuropathic pain’, ‘gut microbiota’, ‘neuropathic pain mechanism’, ‘neuropathic pain treatment’, ‘microbiota-gut-brain axis’. All types of literature were narrative review, systemic review, randomized controlled trial, comparative study, and article, respectively. Moreover, additional publications were searched from the bibliographies of relevant articles to guarantee an integrated collection. Collectively, we systematically address recent advances regarding the role of gut microbiota in regulating the incidence and progression of NP and attempt to provide a potential therapeutic strategy for alleviating NP.

### Microbiota-gut-brain communication

**Fig. 1:**
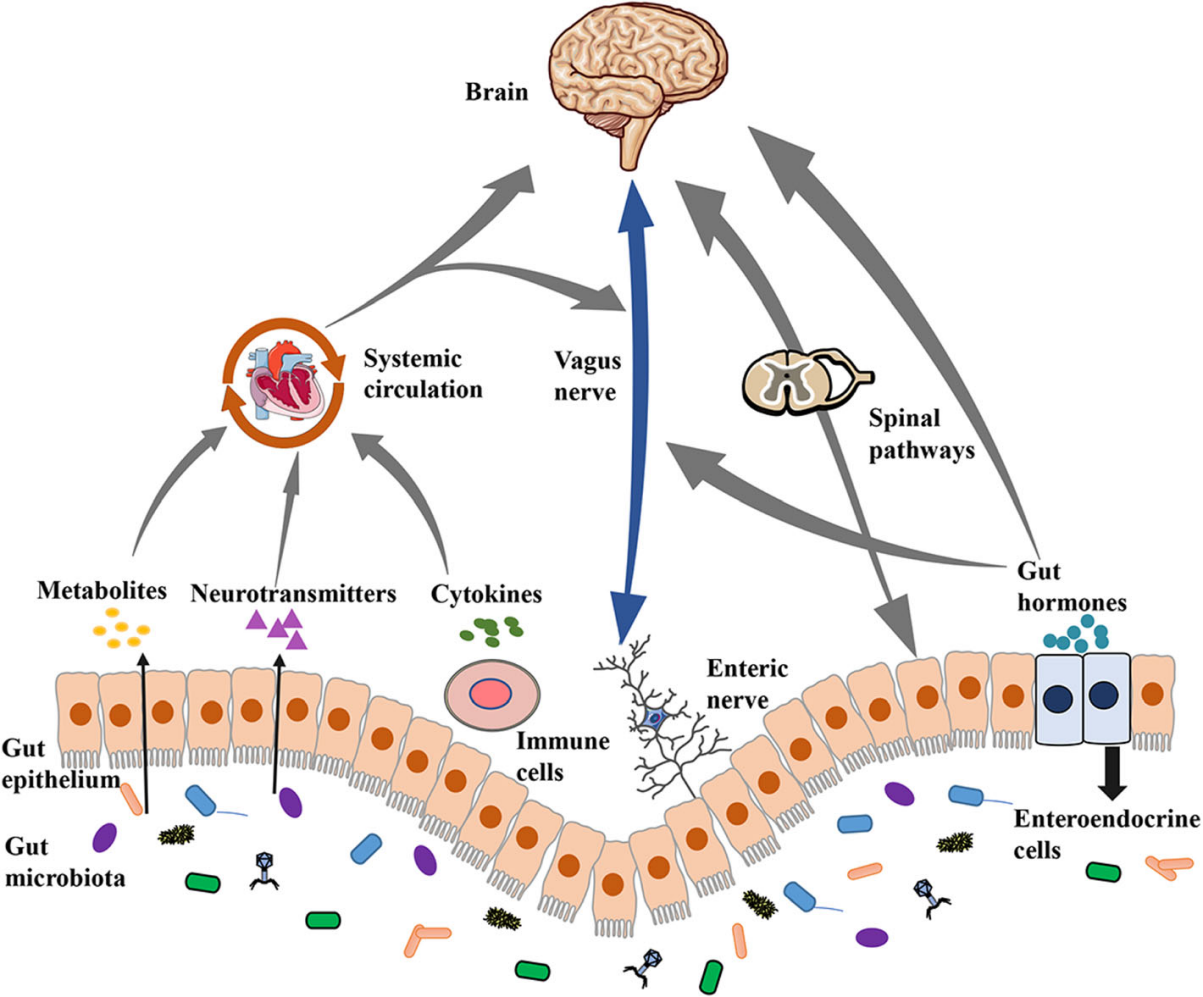
Communication pathways of the microbiota-gut-brain axis. This graph describes the crosstalk of the microbiota-gut-brain axis, which mainly comprise of four modules: metabolic, neural, immune, and endocrine signaling pathways

The bidirectional communication between the gut and brain involves multiple pathways including immune, neural, endocrine, and metabolic routes. Efferent and afferent fibers form a sophisticated reflexive network between the brain and intestine and facilitate interactions within the microbiota-gut-brain axis [14]. This axis comprises various tissues and organs comprising of glands, immune cells, autonomic nervous systems, brain, intestine, and gut microbiota, which crosstalk with a bidirectional manner to maintain homeostasis (Fig. 1). Over past decades, much work has been carried out to define the role of gut-brain interactions in the setting of gastrointestinal tract functional disorders and other disorders that may be related to dysregulated gut-brain communication [15]. Recently, the microbiota-gut-brain axis has drawn increasing attention with the going deep of the medical research. Additionally, microbiota-gut-brain communication implicated in plenty of pathological conditions including Alzheimer’s disease, Parkinson’s disease, depression, and pain, which may directly result in the occurrence of disease by disturbing the balance of the axis.

### Role of gut microbiota in neuropathic pain

#### Microbiome-associated immune signaling

##### Chemokines and cytokines

Abundant literature illustrated that the peripheral and central nervous systems impair triggers cascade of reactions, and thereby construct the chemokine–cytokine architecture, which is closely correlated to the occurrence of neuroinflammation [16, 17]. The alteration of chemokine–cytokine network results in the peripheral sensitization associated with peripheral nociceptive processing [18]. Similarly, glial cells in the spinal dorsal horn (SDH) triggered by inflammatory molecules directly participate in the development of NP via central sensitization [16, 19]. Pro-inflammatory cytokines and chemokines, such as tumor necrosis factor-alpha (TNF-α) and interleukin-1β (IL-1β), produced by various types of cells including immune cells. These molecules form the main mechanism that promotes the neuro-immune communication [20] and elicits the spontaneous discharges by directly sensitizing A- and C-fibers, which is associated with allodynia and hyperalgesia following nerve injury [21, 22]. Related studies showed that the inhibition of upregulated chemokines and their receptors in the peripheral and central nervous systems effectively relieves NP [23]. Thus, cytokines and chemokines play a vital role in processes causing NP. Consistently, numerous drugs have been designed to block cytokine and chemokine signaling; nevertheless, preclinical and clinical studies assessing these receptor antagonists are limited.

The alteration of gut microbiota and its metabolites is related to intestinal dysfunction and systemic immune responses that are generally accompanied the release of numerous pro-inflammatory mediators by immune and glial cells. Pathogen-associated molecular patterns (PAMPs) derived from gut microbiota contain a remarkable array of components, including lipopolysaccharides (LPS) and peptidoglycan (PGN), which are released locally, enter the bloodstream and interact with pattern recognition receptors (PRRs) [24, 25]. Also, PAMPs are key mediators of peripheral sensitization of chronic pain [26]. Clinically, chemotherapy-induced destruction of the intestinal epithelial barrier causes intestinal flora to translocate and release harmful endogenous substances. These substances stimulate PAMPs and PRRs of host antigen-presenting cells and provoke the generation of pro-inflammatory mediators, which constitute an important component of the pathogenesis of CIPN [27]. Shen et al. revealed that the aggregation of macrophages and cytokines in dorsal root ganglion (DRG) are considerably reduced after the administration of oxaliplatin compared with water, demonstrating that the inflammatory response caused by gut microbiota was suppressed in mice treated with antibiotics [12]. Of note, *Lactobacillus fermentum* KBL374 and KBL375 can prominently increase the production of the anti-inflammatory cytokine IL-10, with subsequently inhibiting the expression of other pro-inflammatory cytokines and chemokines [28–30] (Table 1). Also, results from other strains of lactobacillus suggested that these bacteria mediate immunosuppression by decreasing the production of pro-inflammatory cytokines. These results documented that alteration of gut microbiota could lead to the up-regulation and down-regulation of cytokines and chemokines at the same time, which may affect the occurrence of NP. Due to the absence of specific biomarkers for diagnosing NP to date, further studies are needed to research gut microbiota dysbiosis and determine whether gut microbiota influences the development of NP via the induction of immune responses with these pro-inflammatory mediators. In addition, studies also could identify microbiota subgroups that play the greatest role to obtain better efficacy.

**Table 1:** Microbial mediators/species associated with the underlying mechanisms of neuropathic pain

Microbial mediators or species	Function	Potential mechanisms related to neuropathic pain	References
LPS	Activate TLR4	TLR4 contributes to neuropathic pain	Kawai et al. (2010) [31]
	Activate TRPA1 in a TLR4-independent and membrane-delimited manner	The activation of TRPA1 can evoke nociceptive neurons depolarization and firing	Meseguer et al. (2014) [32]
	Activate TRPV1-mediated capsaicin responses via TLR4	Capsaicin responses lead to the excitation of nociception neurons	Diogenes et al. (2011) [33]
Bacterial flagellin	Activate TLR5	TLR5 facilitates the release of pro-inflammatory mediators	Kawai et al. (2010) [31]
	Activate TLR5	TLR5-mediated A-fiber blockade inhibits mechanical allodynia	Kawai et al. (2010) [31]
Indole, LPS	Regulate the secretion of GLP-1	GLP-1 is associated with pain hypersensitivity	Chimerel et al. (2014) [34], Nguyen et al. (2014) [35]
SCFAs	Activate microglia	The activation of microglia leads to pain hypersensitivity	Borre et al. (2014) [36]
	Stimulates the production of PYY and GLP-1 in a FFAR2 and FFAR3 receptors dependent way	GLP-1, PYY are associated with pain hypersensitivity	Tolhurst et al. (2012) [37], Psichas et al. (2015) [38], Lin et al. (2012) [39]
PUFAs	An endogenous agonist of TRPV4	The activation of TRPV4 leads to peripheral hypersensitivity	Cenac et al.(2015) [40]
Bacteria-derived secondary bile acids	Facilitates the release of GLP-1 and PYY via TRG5	GLP-1, PYY are associated with pain hypersensitivity	Ullmer et al. (2013) [41], Thomas et al. (2009) [42], Katsuma et al. (2005) [43]
*Lactobacillus fermentum* KBL374 and KBL375	Increase IL-10 secretion while decrease pro-inflammatory mediators secretion	IL-10 is associated with anti-inflammatory effects	Jang et al. (2019) [28]
*Bacteroides fragilis*	Facilitate the polarization of macrophages to M1 type and enhance their phagocytosis	M1 macrophages can release pro-inflammatory cytokines and express TLRs	Deng et al. (2016) [44]
*Escherichia coli, Lactobacillus*	Synthesize GABA	GABA can reverse allodynia in the neuropathic pain model	Zhao et al. (2017) [45], Wu et al. (2017) [46]
*Escherichia coli*, *Streptococcus* spp., and *Enterococcus* spp.	Produce 5-HT	5-HT serve as a special regulator in NP	Guo et al. (2019) [11]
Corynebacterium glutamicum	Produce glutamate	Glutamate can affect hyperalgesia in neuropathic pain models	Nakayama et al. (2018) [47], Yang et al. (2017) [48], Persicke et al. (2015) [49]
*Lactobacillus, Peptostreptococcus, Clostridium* sporogenes	Generate AHR ligands derived from tryptophan	Act directly on astrocytes through AHR and limit inflammation and neurodegeneration	Zelante et al. (2013) [50], Wlodarska et al.(2017) [51], Dodd et al. (2017) [52]
DSF formulation	Attenuate inflammatory signals	Neutralize the influence of upregulation of TRPV1 and TRPV4 induced by paclitaxel	Castelli et al. (2018) [53]

*Abbreviations*: *LPS* lipopolysaccharide, *TLR* Toll-like receptor, *TRPA1* transient receptor potential cation channel, subfamily A, member 1, *TRPV1* transient receptor potential cation channel, subfamily V, member 1, *TRPV4* transient receptor potential cation channel, subfamily V, member 4, *SCFAs* short-chain fatty acids, *PUFAs* polyunsaturated fatty acids, *GABA* γ-aminobutyric acid, *GLP-1* glucagon-like peptide 1, *PYY* peptide YY, *FFAR* free fatty acid receptor, *TRG5* G protein-coupled bile acid receptor, *AHR* aryl hydrocarbon receptor, *IL-10* interleukin-10

##### Toll-like receptors (TLRs)

**Fig. 2:**
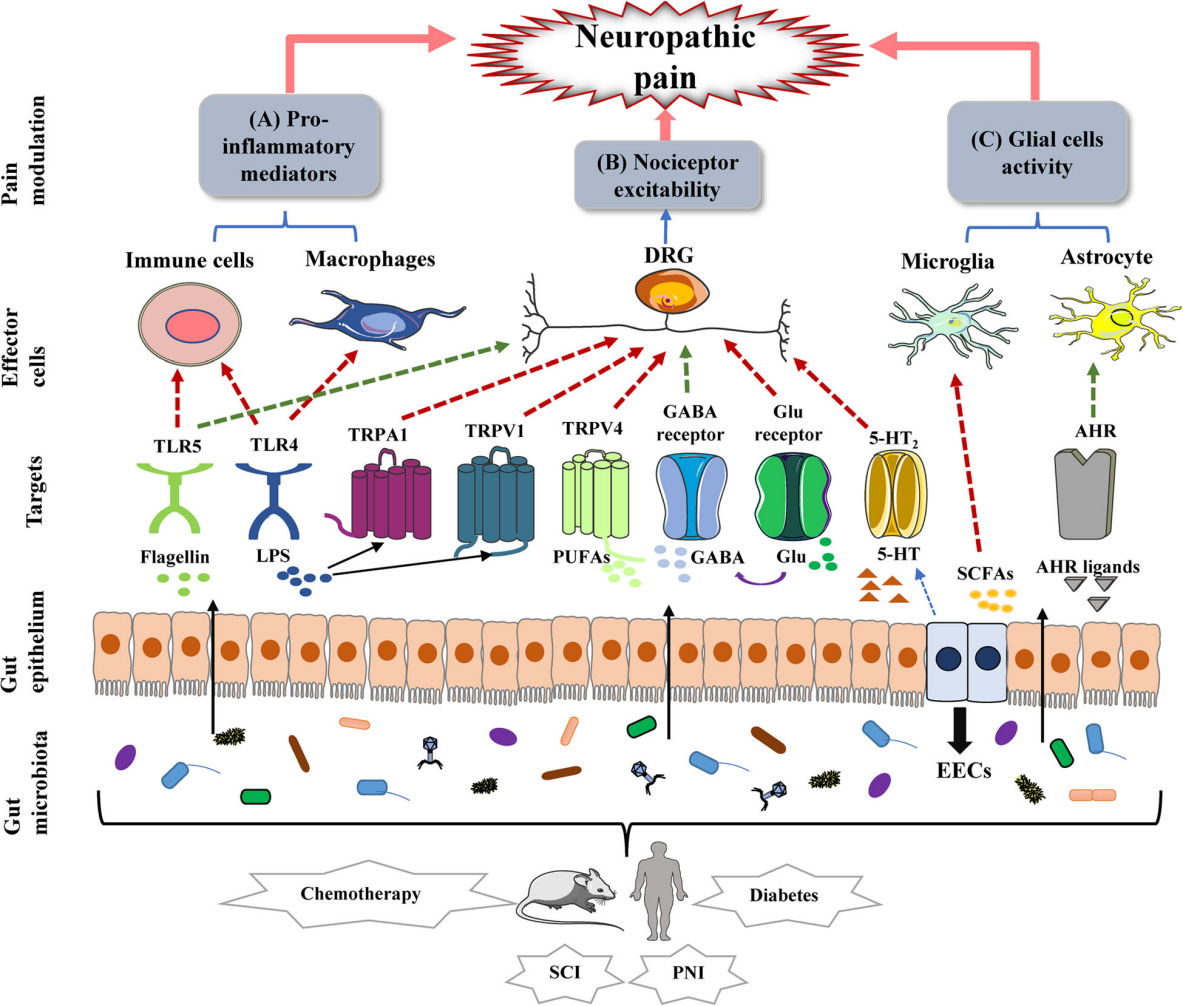
The potential role of gut microbiota in neuropathic pain. Gut microbiota-derived mediators participate in the modulation of neuropathic pain through three routes: **a** LPS and flagellin act on immune cells and macrophages through TLR, and lead to the release of pro-inflammatory mediators; **b** Different mediators alter nociceptor excitability via diverse receptors expressed on DRG neurons; **c** Metabolites regulate glial cells activity directly or through AHR. The red dotted line represents exacerbating pain and the green one represents alleviating pain. **Abbreviations:** DRG, dorsal root ganglion; TLR, Toll-like receptor; TRPA1, transient receptor potential cation channel, subfamily A, member 1; TRPV1, transient receptor potential cation channel, subfamily V, member 1; TRPV4, transient receptor potential cation channel, subfamily V, member 4; GABA, γ-aminobutyric acid; Glu, glutamate; AHR, aryl hydrocarbon receptor; LPS, lipopolysaccharide; PUFAs, polyunsaturated fatty acids; SCFAs, short-chain fatty acids; ECCs, enteroendocrine cells; SCI, spinal cord injury; PNI, peripheral nerve injury

Broadly distributed on most immune cells and other cell types, TLRs are a member of PRRs that activate innate and adaptive immune systems [26]. TLRs are categorized into two types, including extracellular and intracellular receptors. The former recognizes PAMPs, such as LPS derived from microbiota, whereas the latter recognizes the nucleic acids of viruses, bacteria, and hosts [54, 55]. When activated, their downstream signaling pathways contribute to the sustained production of numerous immune pro-inflammatory mediators [56]. TLR4 may also play an indispensable role in the occurrence of NP. Previous studies illustrated that hyperalgesia and allodynia in TLR4-mutant mice are significantly reduced in chemotherapy and nerve injury-induced NP models [57, 58]. Different TLRs sense different PAMPs. For example, TLR2 detects PGN and lipoteichoic acids, TLR4 binds LPS and TLR5 recognizes bacterial flagellin [31] (Table 1). A series of TLR4-mediated signaling pathways are triggered after TLR4 recognizes LPS and promotes the activation of glial cells. When stimulated by flagellin, TLR5 also facilitates the release of pro-inflammatory mediators from immune cells, which contributes to the development of NP [25]. Interestingly, TLR5 activation simultaneously also results in the blockade of sodium currents mainly in A-fibers of mouse DRG and successfully inhibits mechanical allodynia following chemotherapy, diabetic neuropathy, and nerve injury [59] (Fig. 2). Thus, we conclude that gut microbiota plays a dual role by acting on the TLR-mediated pain-related conduction pathways. Due to the vague definition of “harmful”, it might be excessively simplistic to remove some pathogens from gut microbiota, to improve the pain condition.

##### Macrophages

Numerous macrophages reside in the gastrointestinal tract and play a critical role in regulating body function and maintaining homeostasis. According to different functional phenotypes, macrophages can be polarized into two types: M1 and M2. M1 is characterized by a high expression of pro-inflammatory cytokines and receptors, while M2 is characterized by a good deal of anti-inflammatory cytokines [60–62]. There is a bilateral communication between macrophages and nociceptors, which is embodied in macrophages releasing pro-inflammatory mediators to ‘talk to’ nociceptors while macrophages showing ‘listening to’ neuropeptides and chemokines secreted by nociceptors [63]. Many experimental NP animal models exhibit the activation and accumulation of macrophages. Specific inhibition or consumption of macrophages in these models can effectively prevent pain hypersensitivity [64–67], demonstrating that macrophage is an essential regulator of NP (Fig. 2). It is reported that a new strain of *Bacteroides fragilis* could facilitate the polarization of macrophages to M1 type and enhance their phagocytosis [44]. Accumulating evidence suggested that when stimulated by gut microbiota dysbiosis, M1 macrophages release pro-inflammatory cytokines and express TLRs, thus further enhancing neuron-macrophage communication through various pathways. However, a recent study elucidated that gut microbiota could trigger cathepsin K secretion and then induce TLR4-dependent M2 macrophage polarization [68], which may potentially promote anti-inflammatory responses. Grounded on these studies, we conclude that gut microbiota might also play a dual role in macrophage polarization. Given that macrophage polarization and activation is altered in response to the environment [69], how to subtly guide gut microbiota to be more inclined to inhibit the differentiation of M1 macrophages or induce the transformation from M1 to M2 may become a potential treatment to improve NP conditions. Along this direction, the development of an inducing agent with extensive action on microbes represents a significant advance in the goal to specifically block the damage caused by macrophages in NP while maintaining their phagocytic function.

#### Microbiome-associated neural signaling

##### Neurotransmitters

Pain perception involves a variety of neurotransmitters, which can be mainly divided into inflammatory mediators and noninflammatory mediators [70]. The most specific of these neurotransmitters are glutamate and GABA, which are the most widely distributed excitatory and inhibitory neurotransmitters in the body, respectively. Both host and bacteria can convert glutamate to GABA [71]. Some previous studies reported that agents promote the release of GABA by activating GABA receptors, thus effectively relieving trigeminal and diabetic-related NP [72, 73]. Braz et al. demonstrated that GABAergic precursor cell transplantation can reverse allodynia in a mouse NP model and propose transplantation as a therapeutic option in various NP-related models [74, 75]. Furthermore, both the increase of glutamate and the administration of glutamate release inhibitors are sufficient to affect hyperalgesia in animal models [76, 77]. Recently, it has been confirmed that some environmental bacteria strains employed in food fermentation can produce glutamate [47–49]. Also, several strains of bacteria, such as *Escherichia coli* [45], and *Lactobacillus* [46], synthesize GABA (Table 1, Fig. 2). Excitingly, the probiotic Escherichia coli strain Nissle 1917 (EcN) can generate a GABA-related analgesic lipopeptide that inhibits downstream responses caused by nociceptor activation after crossing the intestinal epithelial barrier [78]. In summary, glutamate and GABA in the gut are linked to abundant signaling pathways that modulate pain conditions, regulate the release of pro-inflammatory cytokines, and sense or inhibit afferent innervation of the gastrointestinal tract [79]. However, the host itself also produces GABA. Thus, which of these two sources of GABA predominantly stimulates intestinal neurons and the vagus nerve and ultimately plays a greater role in NP remains unknown.

Serotonin (5-HT), as an important neurotransmitter, could effectively modulate the nociceptive response and serve as a special regulator in NP. When 5-HT acts on its receptors, 5-HT_1_ receptor activation create a hyperpolarizing effect; while 5-HT2 and 5-HT3 activation leads to primary nociceptive neurons depolarized in DRG [80] (Fig. 2). Ji et al. found the activation of the 5-HT_2c_ receptor in the basolateral amygdala facilitates activities in NP-associated central nucleus [81]. Correspondingly, 5-HT_2c_ receptor knockdown contributes to the reduction of NP-related behaviors [82]. More than 90% of 5-HT in the body is synthesized by enteroendocrine cells (EECs) and a growing body of literature reveals that the microbiota is correlated with the host level of 5-HT. Notably, 5-HT can be generated by several strains of bacteria, including *Escherichia coli*, *Streptococcus* spp., and *Enterococcus* spp. [11] (Table 1), but whether gut microbiota can produce 5-HT by de novo remains unknown. Interestingly, 5-HT is reported to be a structural analog of auxins of *Escherichia coli*, *Rhodospirillum rubrum*, and *Enterococcus faecalis,* and activates the growth of these bacteria. Therefore, it might be a hot spot to investigate whether the microbes are able to influence the host 5-HT biosynthesis, and thereby reverse the colonization and development of special microbiota in the intestine [83]. In a word, these findings suggest that the alteration of the microbes may make a difference in the nociception, which is potentially involved in the progression of NP. Though the mechanisms of these neuroactive molecules referred to NP induction and the production of neurotransmitters affected by gut microbiota being far from explicit, it is no denying that gut microbiota is concerned with NP pathogenesis through neurotransmitter routes.

##### Transient receptor potential (TRP) channels

TRP channels are ion channel family members and they are widely expressed on primary afferent nociceptors in DRG. TRP channels act as sensors that convert mechanical, chemical, and thermal stimuli into an inward current [84–86]. TRPA1 and TRPM8 are considered as cold transducers that dominantly mediate cold allodynia [87–89], and experimental results indicated that the administration of their specific inhibitors could alleviate cold hypersensitivity induced by physical nerve injury or chemotherapy [87, 90, 91]. Through altering cell-specific expression patterns, TRPV1 upregulates its expression in DRG [92] and then elicits thermal and chemical hyperalgesia [93]. However, there is a large gap in the understanding of endogenous pro-nociceptive agonists that could activate these channels in pain-related diseases. As a toxic byproduct of bacterial lysis, LPS evokes nociceptive neuron depolarization and firing [7, 33, 94] via the activation of TRPA1 in a TLR4-independent and membrane-delimited manner, supporting the role of TRPA1 in NP [32]. On the other hand, LPS also activates TRPV1-mediated capsaicin responses via TLR4, including intracellular calcium accumulation and inward currents [33], and induces the activation of nociception neurons. Also, polyunsaturated fatty acids (PUFAs) are intestinal microbial metabolites and endogenous agonists of TRPV4 that leads to peripheral hypersensitivity after TRPV4 activation [40, 95] (Fig. 2). On the contrary, the DSF formulation, a high concentration probiotic formulation, attenuates inflammatory signals, thereby neutralizing the upregulation of TRPV1 and TRPV4 induced by paclitaxel. Thus, the DSF formulation is a valid adjuvant agent for inhibiting CIPN [53] (Table 1). Currently, the effect of gut microbiota on ion channels is more concentrated on intermediate media. For example, microbial metabolites act as endogenous agonists of ion channels. Whereas, whether a direct interaction occurs between ion channels and microbes remains unclear.

##### Microglia

Microglial cells are macrophage-like and quiescent immune cells in the CNS, which modulate homeostasis in the spinal cord and brain. Although the precise mechanism of microglia activation in the development of NP has not been fully illustrated, compelling evidence indicates that microglia plays a significant cellular role in the process. In spinal cord injury (SCI) and CIPN models, continuous and massive activation of microglia is widely observed, while a decrease in microgliosis is noted after intrathecal injection with minocycline, contributing to alleviate mechanical allodynia [96–98]. The morphology of microglia within the spinal cord undergoes dramatic changes following increased expression of microglial markers, such as CD11b and Iba1, representing microglial activation after peripheral nerve injury (PNI) [99]. It is well documented that the primary sensory neurons would release microglial activators and associated-signaling molecules are upregulated after PNI, both of which are competent to elicit microgliosis and microglial activation [100]. Additionally, TNF-α and IL-1β, two major pro-inflammatory cytokines, are released and produced by microglia, causing pain conditions through various regulatory mechanisms [101]. Recent emerging evidence has confirmed that microglial activation both in SDH and many brain regions leads to changes in synaptic structure and function and pain hypersensitivity following PNI. Nevertheless, the mechanism by which microglia in these brain regions are activated remains unknown given the long distance from the injured peripheral nerves to the brain [102].

Emerging evidence indicated that the temporal absence of gut microbiota could severely alter the characteristics of microglia. A complex gut microbiota conduces to maintain microglia homeostasis; otherwise, the lack of complex microbiota results in defective microglia. No microglial alteration occurs when microbe-associated molecular patterns are not recognized by various TLRs, demonstrating that microglia may be affected in a microbial-dependent manner [103]. Bacterial products or metabolites such as short-chain fatty acids (SCFAs) serve as a crucial molecule in the maturation and activation of microglia (Fig. 2). These molecules can be translocated from the gut mucosa to the circulatory system and cross the blood-brain barrier (Table 1). Of note, the losing of input signals derived from microbiota in mature microglia can lead to the reacquisition of an immature status. However, the phenotype of microglia can be reversed with the recolonization of complex microbiota in the intestine, which profoundly reveals the significant plasticity of gut microbiota-microglia connection [103]. However, just as the mechanism by which distant microglia cells are activated in the brain remains unclear, the mechanism by which the microbe remotely affects microglia cells in CNS should be explored. We identified that gut microbiota plays an essential role in microglia-mediated signaling pathways in NP.

##### Astrocyte

In the CNS, astrocytes are the dominating population of glia, accounting for approximately 20–40% [104]; these cells supply metabolic support to neurons and maintain glutamate and electrolyte homeostasis [105–107]. Mounting evidence suggests the key modulator of astrocytes in the pathogenesis of pain, especially NP after nerve injuries. First, it has been reported that pain hypersensitivity following PNI in rodents is linked to astrocyte hypertrophy in SDH. In mouse models, it shows that the NP would be ameliorated by suppressing the proliferation of astrocytes [108, 109]. Second, abundant studies elucidate that astrocyte-derived mediators could produce pain hypersensitivity [109]. For instance, the overexpression of C–C motif chemokine 2 in astrocytes results in increased hyperalgesia in mice [110]. Third, recent research found that stimulating astrocytes by transient optogenetic leads to mechanical allodynia as soon as 1 hour after the stimulus in naive rats, demonstrating that astrocyte activation completely drives the occurrence of pain [111]. Collectively, according to the breadth of published literature, we reason out that astrocytes a key driver of NP.

Astrocyte activation is influenced by many factors from inside and outside the CNS [112]. Recently, an emerging study reports a new signaling pathway wherein gut microbiota and environmental cues are integrated to modulate astrocyte activity via circHIPK2 [113], which inhibits astrocyte activation [114]. In addition, dietary tryptophan metabolized by gut microbiota can act directly on astrocytes through aryl hydrocarbon receptors (AHR), limiting inflammation and neurodegeneration in the CNS and providing neuroprotective effects [115, 116] (Fig. 2). Meanwhile, microbial metabolite signaling also regulates the production of transforming growth factor-α (TGFα) and vascular endothelial growth factor-B (VEGF-B) via microglial AHR, further impacting pro­inflammatory activities of astrocytes [116]. Notably, derived from tryptophan, AHR ligands are generated by certain types of bacteria, including *Lactobacillus* [50], *Peptostreptococcus* [51], and *Clostridium sporogenes* [52] (Table 1). On the other hand, TGFα produced by microglia facilitates axon regeneration and increases neuronal survival by inducing astrogliosis and neuroprotective factor generation in SCI models [117, 118]. Thus, we conclude that microglial TGFα promotes salutary astrocyte activities. Currently, the use of commensal bacteria to control TGFα–ErbB1 signaling via AHR has been proposed as an alternative strategy for treating SCI [119]. Therefore, targeting of AHR is likely to establish a microbiota-microglia-astrocyte-oriented treatment for NP.

##### Enteric glia

In the enteric nervous system, enteric glial cells are a unique community of peripheral glial cells related to neurons. Enteric glia takes part in neurotransmission by producing and modulating neurotransmitters, and many of these delivery systems are correlated with the excitability of nociceptors [120]. New data demonstrated that some microbial roles are closely associated with the function and development of enteric glia [121], and the formation of the mucosal enteric glial cell network is synchronized with gut microbiota maturation [122, 123]. Moreover, the introduction of a group of normal gut microbiota can restore the population of impaired mucosal glia [123]. Additionally, enteric glia and astrocytes exhibit morphological and functional similarities, potentially indicating a similar role in pain signaling. Anatomically speaking, enteric glial cells are much closer to intestinal flora than glia in the CNS. Given a lack of understanding of how pain signaling communication occurs between gut microbiota and glia in the CNS due to the significant distance, we hypothesize that enteric glial cells are both a structural and functional mediator of this process. Consequently, adjusting gut microbiota to trigger alterations in these will likely contribute to the identification of a novel treatment for NP.

#### Microbiome-associated endocrine and metabolic signaling

As the gastrointestinal tract is the largest endocrine organ in the human body, gut hormones produced by the enteroendocrine system have a wide range of targets both within and outside the intestinal lumen. To date, several types of EECs have been identified, and all of them are sensory cells [124]. Multiple pleiotropic gut hormones released from EECs are involved in pain modulation, including glucagon-like peptide 1(GLP-1), neuropeptide Y(NPY), and peptide YY(PYY). Previous studies reported that the administration of a GLP-1 analog, such as exendin-4 [125] and the orthosteric agonist of GLP-1 morroniside [126] could alleviate pain hypersensitivity. NPY, acknowledged as a promising target for the treatment for NP for a long time, is widely expressed in the central and peripheral nerve systems, such as enteric neurons and primary afferent neurons [127]. Another member of the neuropeptide family, PYY is exclusively expressed by EECs [127] and has been demonstrated its involvement in the regulation of somatic and visceral pain sensitivity [128]. At present, there is still a large gap in the research on the relationship between EECs with their secreted gut hormones and NP conduction. Given that EECs have a long lifespan [129], they are potentially integrated into the pain-related signaling network involving the immune and nervous systems. Thus, these gut hormones are likely not only endocrine mediators but also immune and neural mediators.

Given the direct dialogue between EECs and gut microbiota at the enteroendocrine interface, their interaction influences gut hormone metabolism. Strikingly, bacterial metabolites directly activate the overwhelming majority of L cells in the distal intestine. For example, the G protein-coupled bile acid receptor (TGR5) distributed on L cells is activated by bacteria-derived secondary bile acids, thus facilitating the release of GLP-1 and PYY from peripheral [41–43]. Additionally, bacterial LPS and the indole produced by bacteria regulate the secretion of GLP-1 [34, 35]. Furthermore, SCFA signaling promotes the generation of PYY and GLP-1 in a free fatty acid receptor 2 ( FFAR2 ) and FFAR3 receptor-dependent manner [37–39] (Table 1). On the other hand, gut microbiota also impacts bile acid metabolism in the host [130]. When bile acids bind to TGR5 expressed in macrophages and primary sensory neurons, two dramatically different outcomes are noted. The activation of neurons in DRG leads to hyperexcitability in a TRPA1-dependent manner, while activation of peripheral macrophages contributes to analgesia [131, 132]. Taken together, through its metabolites and its influence on host metabolism, gut microbiota has established a microbiota-endocrine-metabolic system. Although a considerable portion of gut hormones secreted by EECs are related to pain, more direct preclinical, and clinical studies indicating that hormone molecules participate in signaling involved in the pathogenesis of NP are lacking. To a large extent, gut microbial metabolites potentially participate in NP development through immune and neural signaling pathways, but the existence of an endocrine-metabolism-mediated mechanism also requires further research.

Overall, gut microbiota serves as the intersection of immune, neural, endocrine, and metabolic signaling pathways and has become an intense focus of research. Based on the exciting results in neuroscience over recent years, gut microbiota undoubtedly facilitates the formation of complex and enormous networks and thereby results in the occurrence and development of NP as a pivotal and systematic modulator.

### Potential therapeutic strategy

#### Probiotics and antibiotics

**Fig. 3:**
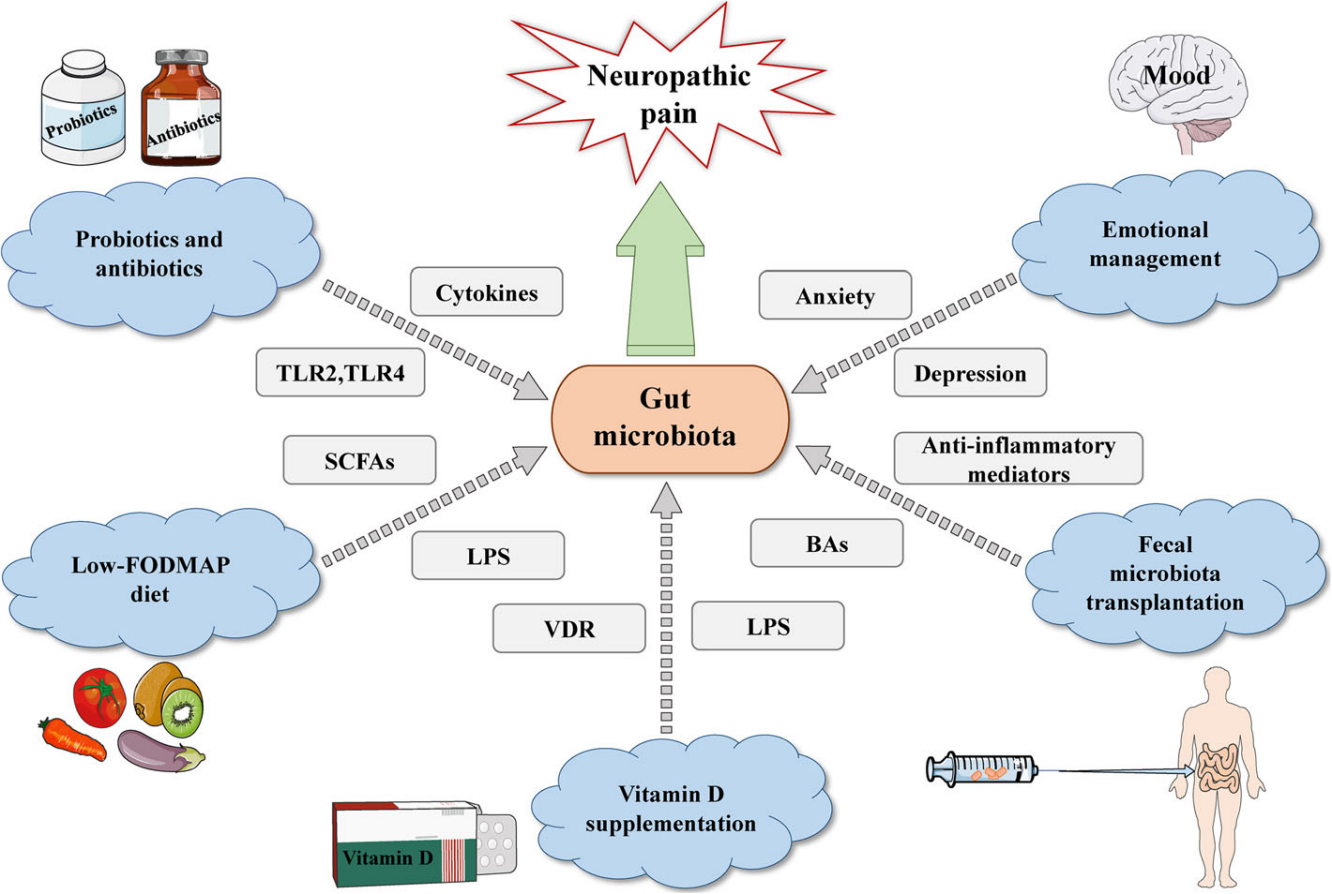
The underlying therapeutic strategy for neuropathic pain through targeting of gut microbiota. There are primarily five therapeutic regimens consisting of probiotics and antibiotics, fecal microbiota transplantation, low-FODMAP diet, vitamin D supplementation, and emotional management for effectively relieving NP. The grey dotted line symbolizes potential mediators/influence factors. **Abbreviations:** TLR, Toll-like receptor; BAs, bile acids; LPS, lipopolysaccharide; SCFAs, short-chain fatty acids; FODMAP, fermentable oligosaccharides, disaccharides, monosaccharides, and polyols; VDR, vitamin D receptor

Given their tremendous potential to alter gut microbiota, probiotics are living bacteria that can provide health benefits, including improved digestion, enhanced immunity, and reduced risk of some diseases [133, 134]. Probiotics alleviate irritable bowel syndrome (IBS), inflammatory bowel disease, and other intestinal dysfunctions. Previous studies suggested that visceral hypersensitivity is improved after the consumption of probiotics in animal models [135]. For instance, VSL#3 and *Lactobacillus paracasei* reverse hyperalgesia and allodynia during colorectal distention [136, 137]. Moreover, probiotics impact the production of cytokines and the expression levels of TLR2 and TLR4, thus modulating immune system activity (Fig. 3). Therefore, probiotics may serve as an inhibitor in immune signaling transmission associated with NP. Despite the view proposed that probiotics affect the nerve function of the gut, there is little work to explicitly and directly prove the veracity of this claim. Shen and colleagues illustrated mechanical hyperalgesia is reduced both in mice preprocessed with antibiotics and GF mice in the CIPN model [12]. Since neither probiotics nor pathogenic bacteria exist in GF mice, the therapeutic effect of antibiotics on CIPN cannot be determined or excluded. In summary, probiotics and antibiotics may change the complexity or activity of microbiota via different mechanisms, but both agents potentially relieve pain in animals and humans. Despite promising findings reported for probiotic and antibiotic therapies, their side effects cannot be ignored. However, research remains at an exploratory stage in this field, reminding us of the need to perform more preclinical and clinical work to investigate the role of probiotics and antibiotics treatment in NP based on gut microbiota.

#### Fecal microbiota transplantation (FMT)

Recently, the restoration of gut microbiota to the pre-disease state has become a vital novel treatment, and the new trend of FMT has been used to cure several diseases, such as ulcerative colitis [138] and *Clostridium difficile* infection [139]. Surprisingly, it is reported that a patient diagnosed with fibromyalgia completely recover after the fecal microbiota transplantation [140], making chronic refractory pain-related diseases a potential therapeutic indication of the treatment. Although fibromyalgia is excluded from the diagnosis of NP since 2011, the pathophysiology of fibromyalgia includes small fiber neuropathy, suggesting a partial overlap between the two pathogeneses. The underlying mechanism of FMT that has been proposed to date suggests that it might play a role in pain via immune and metabolic signal transduction. On one hand, certain components of the transplanted healthy flora may evoke accelerated genesis of anti-inflammatory mediators, thereby counteracting the pro-inflammatory mediators. On the other side, FMT acts as a seemingly prominent player in bile acid metabolism, promptly helping to restore secondary bile acid metabolism in patients [141, 142] (Fig. 3). Although the exact treatment mechanism of FMT has not been revealed, its significant potential in the treatment of chronic pain, including NP, cannot be ignored.

#### Low-FODMAP diet

Furthermore, a dietary cure named low-FODMAP (fermentable oligosaccharides, disaccharides, monosaccharides, and polyols) modifies the complex and diverse nature of gut microbiota and its metabolic output. A high-FODMAPs diet results in increasing levels of LPS derived from the microbial community and the imbalance of gut microbiota, whereas a low-FODMAP diet has a lower level of LPS [143]. Therefore, the low-FODMAP diet tends to contribute to protect the intestinal barrier and reduce gut mucosal inflammation by regulating the level of LPS. In addition, some research suggested that the low-FODMAP diet may also lead to the decreased production of SCFAs in the gut [144]. An animal study demonstrated that SCFAs are correlated with abdominal hypersensitivity [145]. Given that a higher concentration of SCFAs is linked to the symptomatology of IBS, reducing SCFAs may be another approach by which this dietary intervention plays its role. Notably, findings are incompatible regarding the impact of the low-FODMAP diet on SCFAs. It found that the concentration of SCFAs makes no difference between the dietary intervention and controls in two randomized controlled trials of IBS [146, 147]. A small part of patients with IBS suffers from refractory and constant pain, manifesting more as a neuropathic process to a large extent, thus making the visceral pain of IBS adherence to the characters of NP at least. Although there is a lack of direct research on NP and the low-FODMAP diet so far, a large number of studies have confirmed the effectiveness of this dietary intervention on curing IBS through potential gut microbiota-related pathways. Logically, the dietary intervention co-implemented with microbe-targeted therapy is likely to be an emerging approach for NP treatment (Fig. 3).

#### Vitamin D supplementation

Vitamin D, a neurotrophic hormone and neuroactive steroid, triggers a series of signal conduction systems including pain. In recent years, more and more studies have confirmed that hypovitaminosis D is an independent risk predictor of diabetic neuropathy progression [148]. Some research also proposed that vitamin D deficiency plays a novel role in the involvement of the mechanistic pathway of multiple sclerosis [149, 150]. It has been known for decades that the lack of vitamin D results in the decreased absorption of calcium, and induces gut stasis. Unfortunately, the abnormality of intestinal motility enhanced gut permeability allowing a growing release and transfer of endotoxins from gut microbiota [150]. In the long term, translocated LPS stimulates the increased production of pro-inflammatory mediators, ultimately causing neuroinflammation and contributing to the development of multiple sclerosis. In addition to impacting on the gut barrier, vitamin D may change the composition of gut microbiota communities via activating vitamin D receptor signaling [151]. Expressed in muscle tissue and CNS, these receptors are associated with innate immune response [152], which has an advantageous effect on keeping homeostasis from disturbance related to neuropathy to some extent [153]. Several previous works illustrated that a high dose of vitamin D supplementation significantly conduces to decrease typical pathogen species and increase the abundance of phylotype of microbes in the gut [154]. Though numerous studies have reported that vitamin D supplementation prevents neuronal degeneration and improves cold allodynia, mechanical, and heat hyperalgesia in the rat models of NP [155, 156], the proven mechanism remains uncertain. Based on these findings, future studies could address more insights on vitamin D and gut microbiota and exploit a novel and promising strategy to treat or prevent NP (Fig. 3).

#### Emotional management

Presently, numerous studies indicated that depression and anxiety play an instrumental role in the occurrence and development of NP. Clinical research showed that certain antidepressants indeed attenuate the symptom of NP. Whereas, there is a lack of animal models to effectively account for the impact of emotional outcomes on NP. Most strikingly, some literature documented that gut microbiota is closely correlated with psychiatry including depression and anxiety [157]. Preclinical studies suggested that depression induced by early life stress/surgical procedures leads to the alteration of gut microbiota [158, 159]. Correspondingly, the modulation of gut microbiota also affects behaviors related to depression. Furthermore, compelling evidence documented that anxiety-like behaviors could be influenced by the altered gut microbiota [160, 161], and gut microbiota also transfers the anxious phenotype in turn [162, 163]. Thus, the microbiota may be an underlying therapeutic target for psychiatry. Achieving favorable emotional management via the manipulation of gut microbiota is conducive to relief NP by controlling pain comorbidities (Fig. 3).

## Discussion and conclusions

As more and more precise instruments have been designed to identify NP, assessment of its prevalence and socioeconomic influence have risen. The incapacity of targeting underlying mechanisms accurately results in a lower cure success rate. Following the concept of the microbiota-gut-brain axis proposed, accumulating attention is concentrated on the role of gut microbiota in NP, which is conceptually appealing and provides an emerging perspective. This review comprehensively summarizes the current research status of gut microbiota involved in regulating the pathogenesis of NP through various signaling pathways and deeply discusses the feasibility and challenges of targeting of gut microbiota for treating NP. As the saying goes “all disease begins in the gut”, the microbiota-gut-brain axis provides a more scientific explanation for illustrating the basic theory. Along with this axis, we integrate the existing elements associated with the mechanism of NP and establish a complicated immune-neural-endocrine-metabolic systemic network.

Currently, inadequate studies could fully clarify the sophisticated principle concerning the relationship between gut microbiota and NP. A diverse array of intermediate constructs a bridge between both, but some phenomena lack rational explanations mechanistically. One question is how the dysfunctional gut microbiota and its derived mediators transfer into DRG and some even cross the blood-brain barrier (BBB) into the CNS. Although some previous studies documented several pieces of evidence to support the capability of gut microbiota to impact on BBB permeability [164, 165], the proven mechanism is still unclear. Logically, we have to admit that gut microbiota is correlative with NP instead of causal and mentioned signaling pathways of NP reinforce each other and act concurrently. In terms of mechanisms, it will be a great success if aiming at part of a matter along the signal transduction pathways contributes to improving NP-related symptoms.

Present therapies of NP are usually curative, and surplus pain is common even during treatment. Several safer, more economical, and less invasive settlements are more adapted to some patients, and thus the therapeutic approach step by step is cautious [166]. In lots of different situations, the complexities and difficulties of individual cases may reveal the need for multimodal and multidisciplinary NP management strategies. Regarding drug therapy, clinicians are required to pore over the efficacy, the adverse effects, as well as any comorbidities [167]. Additionally, the interventional management of NP is fraught with lots of practical challenges and ethical bias [168]. Therefore, the proposed approach such as low-FODMAP diet, vitamin D supplementation, and emotional management are much less risky, operationally easier, and more acceptable psychologically. Excluded traditional drugs and surgery, these emerging treatments are likely to enjoy high popularity. But then again, we must acknowledge that some controversial applications and secondary action exist. Certain previous research claimed that antibiotics improve the condition of NP, while some illustrated antibiotics could result in hyperalgesia [27]. The administration of antibiotics in different dosage and choice, and differences in components of antibiotics lead to distinct dysfunction of gut microbiota deserve to be discussed. As for FMT, though some anaerobic microbes confirmed to be successfully cultured [169] until recently multitude of gut microbiota cannot be cultivated [170]. Furthermore, the side effects of FMT comprise some self-limiting abdominal uncomfortableness, and, rarely, contagious diseases that are difficult to detect by testing [171], which also needs to be alert.

So far concerned, majority studies have focused on the general role of gut microbiota in NP, but the more detailed characterization of the microbiome population, species, and activity in the pain progression and whether gut microbiota can be a biomarker for NP remains unknown. As long as the most beneficial microbial components for a particular clinical status is determined, the difficulty would be to change the microbiota characteristics to replicate this composition as much as possible. The further step will be manipulating gut microbiota more precisely, for instance by bringing in specific microbes to defeat cacoethic strains. Collectively, there being tremendous enthusiasm for the microbiome in academia, targeting gut microbiota has become a rapidly growing therapeutic approach for a wide range of diseases including NP, contributing to facilitating the translation of this finding from bench to bedside.

## Data Availability

All data were included.

## Abbreviations

NP: Neuropathic pain
CIPN: Chemotherapy-induced peripheral neuropathy
DRG: Dorsal root ganglion
TLR: Toll-like receptor
TRP: Transient receptor potential
AHR: Aryl hydrocarbon receptor
TGR5: G protein-coupled bile acid receptor
FFAR: Free fatty acid receptor
PGN: Peptidoglycan
PAMPs: Pathogen-associated molecular patterns
PRRs: Pattern recognition receptors
LPS: Lipopolysaccharide
PUFAs: Polyunsaturated fatty acids
SCFAs: Short-chain fatty acids
ECCs: Enteroendocrine cells
CNS: Central nerve system
TGF-α: Transforming growth factor-α
VEGF-B: Vascular endothelial growth factor-B
GLP-1: Glucagon-like peptide 1
NPY: Neuropeptide Y
PYY: Peptide YY
GF: Germ-free
SCI: Spinal cord injury
PNI: Peripheral nerve injury
BAs: Bile acids
FODMAP: Fermentable oligosaccharides, disaccharides, monosaccharides, and polyols
FMT: Fecal microbiota transplantation
IBS: Irritable bowel syndrome
BBB: Blood-brain barrier
